# MSX2 Induces Trophoblast Invasion in Human Placenta

**DOI:** 10.1371/journal.pone.0153656

**Published:** 2016-04-18

**Authors:** Hao Liang, Qian Zhang, Junjie Lu, Genling Yang, Na Tian, Xiaojie Wang, Yi Tan, Dongmei Tan

**Affiliations:** Laboratory Animal Center, Chongqing Medical University, Chongqing, China; University of Alabama at Birmingham, UNITED STATES

## Abstract

Normal implantation depends on appropriate trophoblast growth and invasion. Inadequate trophoblast invasion results in pregnancy-related disorders, such as early miscarriage and pre-eclampsia, which are dangerous to both the mother and fetus. Msh Homeobox 2 (MSX2), a member of the MSX family of homeobox proteins, plays a significant role in the proliferation and differentiation of various cells and tissues, including ectodermal organs, teeth, and chondrocytes. Recently, MSX2 was found to play important roles in the invasion of cancer cells into adjacent tissues via the epithelial-mesenchymal transition (EMT). However, the role of MSX2 in trophoblastic invasion during placental development has yet to be explored. In the present study, we detected MSX2 expression in cytotrophoblast, syncytiotrophoblast, and extravillous cytotrophoblast cells of first or third trimester human placentas via immunohistochemistry analysis. Furthermore, we found that the in vitro invasive ability of HTR8/SVneo cells was enhanced by exogenous overexpression of MSX2, and that this effect was accompanied by increased protein expression of matrix metalloproteinase-2 (MMP-2), vimentin, and β-catenin. Conversely, treatment of HTR8/SVneo cells with MSX2-specific siRNAs resulted in decreased protein expression of MMP-2, vimentin, and β-catenin, and reduced invasion levels in a Matrigel invasion test. Notably, however, treatment with the MSX2 overexpression plasmid and the MSX2 siRNAs had no effect on the mRNA expression levels of β-catenin. Meanwhile, overexpression of MSX2 and treatment with the MSX2-specific siRNA resulted in decreased and increased E-cadherin expression, respectively, in JEG-3 cells. Lastly, the protein expression levels of MSX2 were significantly lower in human pre-eclamptic placental villi than in the matched control placentas. Collectively, our results suggest that MSX2 may induce human trophoblast cell invasion, and dysregulation of MSX2 expression may be associated with pre-eclampsia.

## Introduction

Favorable development of the embryo after implantation depends on the formation of a functional placenta. During placental development, the growth and invasion of trophoblast cells is affected by strict spatio-temporally expressed regulators [1], and inadequate trophoblast invasion leads to early miscarriage, pre-eclampsia (PE), intrauterine growth retardation, and other clinical diseases [2]. More severely, uncontrolled invasiveness can lead to conversion of normal trophoblast cells to choriocarcinomas. Trophoblast progenitor cells, also called cytotrophoblasts (CTB), originate from the outer layer of the blastocyst, provide nutrients for the embryo, and develop into the fetal portion of the placenta. Moreover, under distinct conditions, CTBs further differentiate into syncytiotrophoblasts (STB) or extravillous cytotrophoblast cells (EVT) [1, 3]. The STB is a multinucleated monolayer located in the outer layer of the villus that comes in direct contact with the maternal blood that reaches the placental surface, and thus facilitates the exchange of nutrients, waste, and gases between the maternal and fetal systems. In humans, the EVT undergoes an epithelial-mesenchymal transition (EMT) [4], initially forming multilayered cell columns that subsequently deeply infiltrate the maternal decidual stroma and blood vessels [5, 6]. Matrix metalloproteinases (MMPs) with collagenase activity, particularly MMP-2 and MMP-9, are important during early embryonic and placental development. The activity of MMPs in the breaching of the extracellular matrix barrier by trophoblasts during embryo implantation and early placental development. Thus, there is considerable evidence that MMPs play essential roles in trophoblast invasion at the fetal-maternal interface [7, 8].

The members of the MSX family of homeobox proteins, comprising MSX1, MSX2, and MSX3, are critical regulators of tissue morphogenesis. In these three members, MSX2 was found to play important roles in the development, growth, and differentiation of various types of cells and tissues, including ectodermal organs, teeth, and chondrocytes [9–11]. Notably, MSX2 is also abnormally expressed in a variety of carcinoma cells, including adenocarcinoma [12], breast cancer [13], and ovarian endometrioid carcinoma [14], in which MSX2 expression is highly correlated with cell invasion levels. Furthermore, the expression patterns of the MSX2 gene during organ development suggest its pivotal role in the EMT. Indeed, in humans, MSX2 induces the development of postnatal mammary glands by promoting EMT [15]. Consistent evidence was found in NMuMG cells, a spontaneously immortalized normal mouse mammary epithelial cell line [16]. Furthermore, MSX2 as the mediator of BMP-4-induced EMT was found in a pancreatic cancer cell line [17].

Several members of the *WNT* gene family were reported which upregulated in the uterine epithelium and stroma of preimplantation uteruses in MSX1/2^d/d^ mice. Furthermore, the canonical Wnt signaling pathway was found to be activated in stromal cells, thereby preventing cell differentiation and creating a non-receptive uterus refractory to implantation [18]. Meanwhile, loss of MSX1/MSX2 expression was shown to be correlated with altered uterine luminal epithelial cell polarity, and to affect E-cadherin/β-catenin complex formation via modulation of Wnt5a expression [19]. Lastly, previous studies indicate that MSX2 plays an important role in mammalian embryonic diapause [20, 21].

While trophoblastic cells and cancer cells exhibit several similar behavioral characteristics, including the mechanism by which they invade adjacent tissues via EMT [5], it is currently unclear whether MSX2 plays a role in regulating trophoblast cell invasion during embryo implantation. Based on the findings described above, we propose that MSX2 might enhance the invasive capacity of trophoblast cells by promoting EMT. To address this hypothesis, we first characterized the expression pattern of MSX2 in both human placental villi and trophoblast-derived cells. Subsequently, we examined the effects of different MSX2 expression levels on the in vitro invasive ability of HTR8/SVneo cells, as well as on the expression levels of β-catenin, vimentin and E-cadherin, to elucidate the role of MSX2 during the EMT process. Lastly, we compared the differential expression patterns of MSX2 in pre-eclampsic and normal placental villi to investigate the role of MSX2 in PE pathogenesis.

## Materials and Methods

### Tissue collection

All sample tissues used for immunohistochemical and western blot analyses were collected, and then washed with ice-cold PBS and subjected to fixation (first trimester: n = 5; second trimester: n = 5) or protein extraction (third trimester: n = 8; PE third trimester: n = 8). Human placental tissues of the first trimester (5 weeks) and the second trimester (15 weeks) were obtained from healthy women who underwent abortion for nonclinical reasons. Term placentas (normal and pre-eclamptic) were collected after cesarean section. PE was defined as new onset of both hypertension (blood pressure ≥ 140/90 mmHg in previously normotensive women) and proteinuria [≥300 mg/24-h urine collection or random urine protein (+)] after 20 weeks of gestation. None of PE patients included in the study was complicated with other diseases. Matching to the control patients, all PE patients were aged 25–33, and delivered between 36 to 40 weeks. All volunteers provided informed written consent to donate their placentas for use in scientific research. Ethical approval was granted by the Ethics Committee of the First Affiliated Hospital of Chongqing Medical University.

### Cell culture, MSX2 overexpression, and RNAi-mediated knockdown of MSX2 expression

The HTR8/SVneo, JEG-3, JAR, and BeWo cell lines were provided by Dr. Hongmei Wang (Institute of Zoology, Chinese Academy of Sciences), and HTR8/SVneo cells were used as a model for trophoblast invasion experiments. HTR8/SVneo and JAR cells were cultured in RPMI 1640 (Hyclone, Logan, UT, USA) containing 10% fetal bovine serum (FBS; Gibco), 100 U/mL penicillin, and 100 μg/mL streptomycin, while JEG-3 and BeWo cells were maintained in DMEM/F-12 medium (1:1) supplemented with 10% FBS, 100 U/mL penicillin, and 100 μg/mL streptomycin. All cells were maintained at 37°C with 5% CO_2_.

For overexpression of MSX2, HTR8/SVneo and JEG-3 cells were grown to approximately 70% confluency in 35-mm dishes and transfected with 3 mg of the Flag-MSX2 plasmid (GeneCopeia, Rockville, MD, USA), using Viafect^™^ Transfection Reagent (Promega, Madison, WI, USA), as recommended by the manufacturer. Cells transfected with empty pFLAG-CMV vector were utilized as a control. For RNAi experiments, HTR8/SVneo and JEG-3cells were transfected with 100 nM MSX2-specific siRNA (Santa Cruz Biotechnology, Dallas, TX, USA) or the control siRNA (a universal negative control; Santa Cruz Biotechnology), using Viafect^™^ Transfection Reagent, according to the manufacturer’s instructions. The transfection efficiency was >50%, as determined using fluorophore-labeled siRNA molecules (Santa Cruz, USA). HTR8/SVneo or JEG-3 cells were then harvested at 24 h post-transfection and subjected to transwell assay, MTT [3-(4,5-dimethylthiazol-2-yl)-2,5-diphenyltetrazolium bromide] assay, and flow cytometry analyses.

### Immunohistochemistry

Tissue samples were fixed in 10% buffered formalin and embedded in paraffin. For immunofluorescence staining analyses, paraffin embedded tissue biopsies were cut into 5 μm sections, dehydrated with xylene and graded alcohol/water mixtures, deparaffinized, rehydrated, and treated with 3% H_2_O_2_ for 10 minutes to inhibit endogenous peroxidase. Antigen retrieval was performed at 95–98°C in citrate buffer [10 mM citrate sodium and 10 mM citric acid (pH 6.0)] for 15 min. After blocking with normal goat serum for 30 min, sections were incubated with rabbit anti-MSX2 (1:50; sc-15396; Santa Cruz Biotechnology) or mouse anti-Cytokeratin 7 (1:200; ab20206; Abcam, Cambridge, United Kingdom) at 4°C for 12–14 h. Sections were then incubated with a rabbit or mouse secondary antibody (Beyotime Biotechnology, Beijing, China) for 1 h and stained using a diaminobenzidine kit (Beyotime Biotechnology, Beijing, China). For negative controls, the primary antibody was replaced with goat serum or IgG from the same species in which the primary antibodies were generated.

### Western blot analysis

HTR-8/SVneo cells were seeded in 60 mm dishes, then transfected with plasmids or siRNA molecules, and cultivated for 24h. Total proteins were extracted using radioimmunoprecipitation (RIPA) buffer (Beyotime) supplemented with phenylmethylsulfonyl fluoride (PMSF; Beyotime), and the protein concentration of each resulting sample was determined using the Bradford assay. Samples containing approximately 50 μg of protein were then separated by 10% sodium dodecyl sulfate-polyacrylamide gel electrophoresis (SDS-PAGE) and transferred to 0.22 mm nitrocellulose membranes. Membranes were probed using primary antibodies specific to MSX2 (1:2,000; sc-15396; Santa Cruz Biotechnology), MMP-2 (1:1, 000; #4022; Cell Signaling Technology, Danvers, MA, USA), MMP-9 (1:1, 000; #3825; Cell Signaling Technology), vimentin (1:3, 000; ab92547; Abcam), E-cadherin (1:6, 000; ab40772; Abcam), β-catenin (1:6, 000; ab32572; Abcam), or glyceraldehyde 3-phosphate dehydrogenase (GAPDH; 1;4, 000; #2118; Cell Signaling Technology). Probed membranes were washed and incubated with horseradish peroxidase-conjugated goat anti-mouse or goat anti-rabbit IgG secondary antibodies (Beijing ZhongShan Biotechnology Co., Beijing, China), and immunoreactive bands were detected using an enhanced chemiluminescence reagent (Thermo Fisher Scientific, Waltham, MA, USA). All experiments repeated at least three times.

### Matrigel cell invasion assays

Matrigel cell invasion assays were performed in Transwell chambers (6.5 mm, Corning Costar, St. Louis, MO, USA). For these analyses, the upper chamber inserts (8 μm pore size) were pre-coated with 50 μL of a 1 mg/mL Matrigel matrix solution and incubated for 4 h at 37°C to allow for gelling. Subsequently, 1 × 10^5^ HTR8/SVneo cells harvested at 24 h post-transfection with siRNA or plasmids were diluted in 200 μL of serum-free medium and seeded in the upper chamber. Meanwhile, the lower well was filled with medium containing 10% FBS. The cells were allowed to invade the lower chamber via penetration of the membrane during a 24 h incubation period at 37°C. Following incubation, invading cells were detached from the bottom of the membrane insert and enumerated microscopically to calculate the invasive potential of the each cell population. The assay repeated three times. Conditional culture medium samples were also collected and subjected to gelatinolytic activity assay analysis, as described below.

### MTT Assay

Cell proliferation was measured by the MTT assay, and performed following the manufacturer’s instructions. HTR8/SVneo cells were plated in 96-well plate at 0.5×10^4^ per chamber and post-transfection with siRNA or plasmids were diluted in 100 μl of serum-free medium. After 20 hours, 10μl MTT (5mg/ml, Zhongshan Corp. Beijing, China) was added to chambers for 4 hours, respectively. 100μl DMSO was then added in each well to dissolve the crystals. The absorbance of the wells were determined using a microplate reader at 490 nm wave length. The experiment was performed in triplicates.

### Gelatin zymography

The enzymatic activities of MMP-2 and MMP-9 in HTR8/SVneo cells were analyzed by gelatin zymography. Appropriate volumes of media were collected after 48 h cultivation and subjected to 0.1% gelatin-8% SDS-PAGE electrophoresis. SDS was removed from the resulting gels by soaking twice in 2.5% Triton X-100 for 30 min, after which the gels were incubated in bath buffer [0.5 M Tris-HCl (PH 7.8), 2 M NaCl, 0.05 M CaCl_2_, and 0.2% Brij 35] for 36 h at 37°C. Following incubation, gels were stained with 0.1% Coomassie blue in 50% methanol and 15% acetic acid, and destained in 10% acetic acid to reveal zones with gelatinase activity.

### Flow cytometry

Cell cycle progression analysis was performed using flow cytometry. Treated cells were collected using trypsin without EDTA, washed twice with PBS, resuspended in 1 mL binding buffer, and fixed by incubating in ethanol (70%) overnight at 4°C. The following day, cells were washed with PBS and the alcohol solution was removed. Cells were then mixed with a fluorochrome solution comprised of propidium iodide (PI 2 mg/mL), RNase A (4 mg/mL), and 0.1% Triton X-100, incubated for 15 min in the dark at room temperature, and analyzed using a flow cytometer (FACSVantage SE, Becton Dickinson and Co., Franklin Lakes, NJ, USA). In addition, the levels of cell apoptosis were evaluated by staining with a FITC-labeled annexin V-specific antibody and PI at room temperature for 15 min. The percentage of early apoptotic cells was calculated by counting the number of cells that were annexin V-positive and PI-negative.

### Quantitative reverse transcription PCR (qRT-PCR) analysis

Total RNA was extracted from cells using TRIzol reagent (Invitrogen, Waltham, MA, USA), according to the manufacturer’s instructions, and quantified spectrophotometrically at 260 nm. Two micrograms of each RNA sample was reverse transcribed using Superscript III reverse transcriptase (Invitrogen). qPCRs were performed in 25-μL volumes comprised of 12.5 μL of SYBR^®^ Premix Ex Taq^™^ II (Tli RNaseH Plus; Takara Bio., Shiga, Japan), 0.4 μM of each primer, 1 μL cDNA template, and ultrapure water. The reactions were performed under the following conditions: 94°C for 10 min, followed by 40 cycles of 94°C for 15 s, 60°C for 30 s, and 72°C for 30 s, and a final extension at 72°C for 10 min to obtain a solubility curve. The primer sets used for quantitative PCR were as follows: MSX2 Forward: 5′-GGAGCGGCGTGGATGCAGGAA-3′, Reverse: 5′-AAGCACAGGTCTATGG AACGG-3′; E-cadherin Forward: 5′-AGCCTCTGGATAGAGAACGCATTG-3′, Reverse: 5′-GGGTGAATTCGGGCTTGTTGTCAT-3′; vimentin Forward: 5′-CGAAAACACCCTGC AATCTT-3′, Reverse: 5′-CTGGATTTCCTCTTCGTGGA-3′; β-catenin Forward: 5′-GAGTG CTGAAGGTGCTATCTGTCTG-3′, Reverse: 5′-GTTCTGAACAAGACGTTGACTTGGA -3′; and GAPDH Forward: 5′-AGCCACATCGCTCAGACAC-3′, Reverse: 5′-TGGACTCCA CGACGTACTC-3′. Relative mRNA expression levels were calculated using the comparative CT (2^−ΔΔCt^) method.

### Statistical Analysis

The bands from Western blotting and gelatin zymography were quantified by MetaView Image Analyzing System (Version 4.50; Universal Imaging Corp., Downingtown, PA). Each experiment was performed at least three times. Data were presented as means ± standard deviations (SD), and differences between results were evaluated by one-way analysis of variance (ANOVA), Student’s *t*-test and Mann Whitney test. All statistical analyses were performed using SPSS software (SPSS Statistics, Inc., Chicago, IL, USA). *P* < 0.05 was considered statistically significant. *, *P <0*.*05*. **, *P <0*.*01*.

## Results

### MSX2 expressed in human placenta villous and invasive extravillous trophoblast

**Fig 1:**
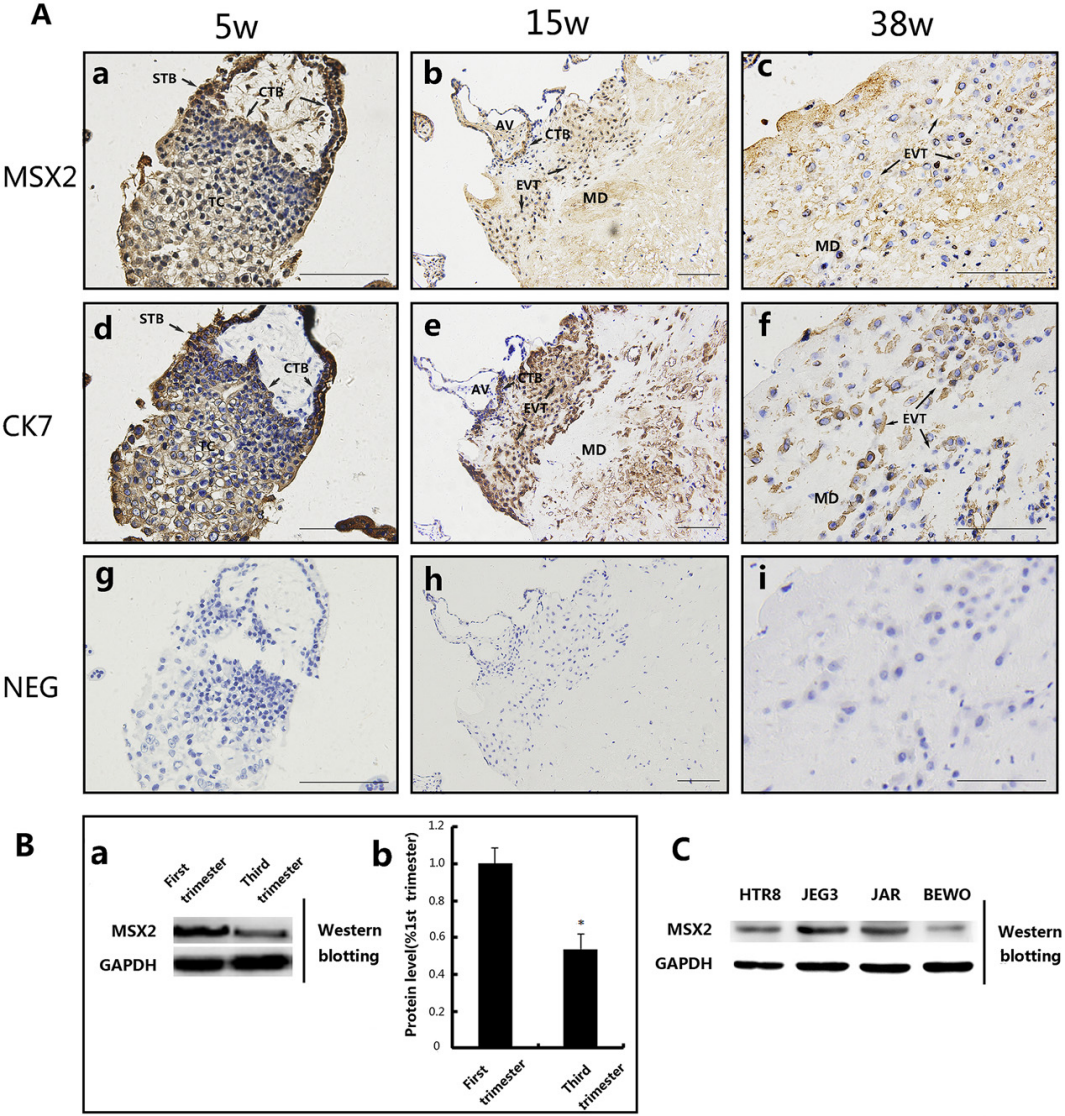
Expression of MSX2 in human placental villi and cell lines. **(A)** Immunohistochemical localization of MSX2 in normal human placental villi at different stages of pregnancy (a) a placental villi from the first trimester. (b) a placental villi from the second trimester. (c) a placental villi from the second trimester. EVTs invaded into the maternal decidua at the second and third trimester immunostained with MSX2 (b,c) and CK7, a marker of EVT (e,f). (g,h,i) negative controls (NEG) in which normal IgG were used in place of primary antibody. CTB: cytotrophoblast cells; STB: syncytiotrophoblast; TC: trophoblastic column; EVT: extravillous trophoblast; AV: anchoring villi MD: maternal decidua; Bar = 200 μm. **(B)** (a) Western blotting of MSX2 in the human placental villi from the first (n = 3) and the third (n = 3) trimesters. GAPDH was used as a loading control(Also C). (b) Three experiments as in a were quantified by measuring the intensity of MSX2 protein bands relative to GAPDH controls (Mann Whitney test; *, *P*<0.05). (**C**) Expression of MSX2 in different trophoblast cell lines determined by Western blotting. HTR8/SVneo: a human invasive extravillous trophoblast cell line derived from immortalized first trimester trophoblast; JEG-3, JAR, and BeWo: human choriocarcinoma cell lines.

To characterize the putative role of MSX2 in placentation, we first examined the location of MSX2 expression in human placental villi by immunohistochemistry. As shown in Fig 1A, MSX2 protein intensely expressed in both the STB, CTBs and trophoblast column(TC) of first trimester placental villi (5 weeks; Fig 1Aa), however predominantly expressed in STB and in invading interstitial EVTs in mid-pregnancy (15 weeks; Fig 1Ab). Meanwhile, in term placentas (38 weeks), the expression of MSX2 protein was detected in villous and EVTs that invaded into the maternal decidua (Fig 1Ac). These findings demonstrate that MSX2 is dynamically expressed in trophoblast lineages throughout gestation. The identification of trophoblast cells were confirmed cytokeratin 7 (CK7) and staining respectively on a separate serial placental sections (Fig 1Ad, 1Ae and 1Af). In addition, expression of MSX2 in term placental villi was significantly lower than that in the first trimester based on Western blotting (Fig 1Ba and 1Bb; *P*<0.05). Furthermore, we examined the expression of MSX2 in human trophoblast cell lines by western blotting MSX2 proteins were present in HTR8/SVneo cells (normal trophoblast cell lines), were high in JEG-3 cells (human choriocarcinoma cell lines) and JAR cells (human choriocarcinoma cell lines), but low in BeWo cells (human choriocarcinoma cell lines) (Fig 1C).

### MSX2 induces invasion in HTR8/SVneo cells

**Fig 2:**
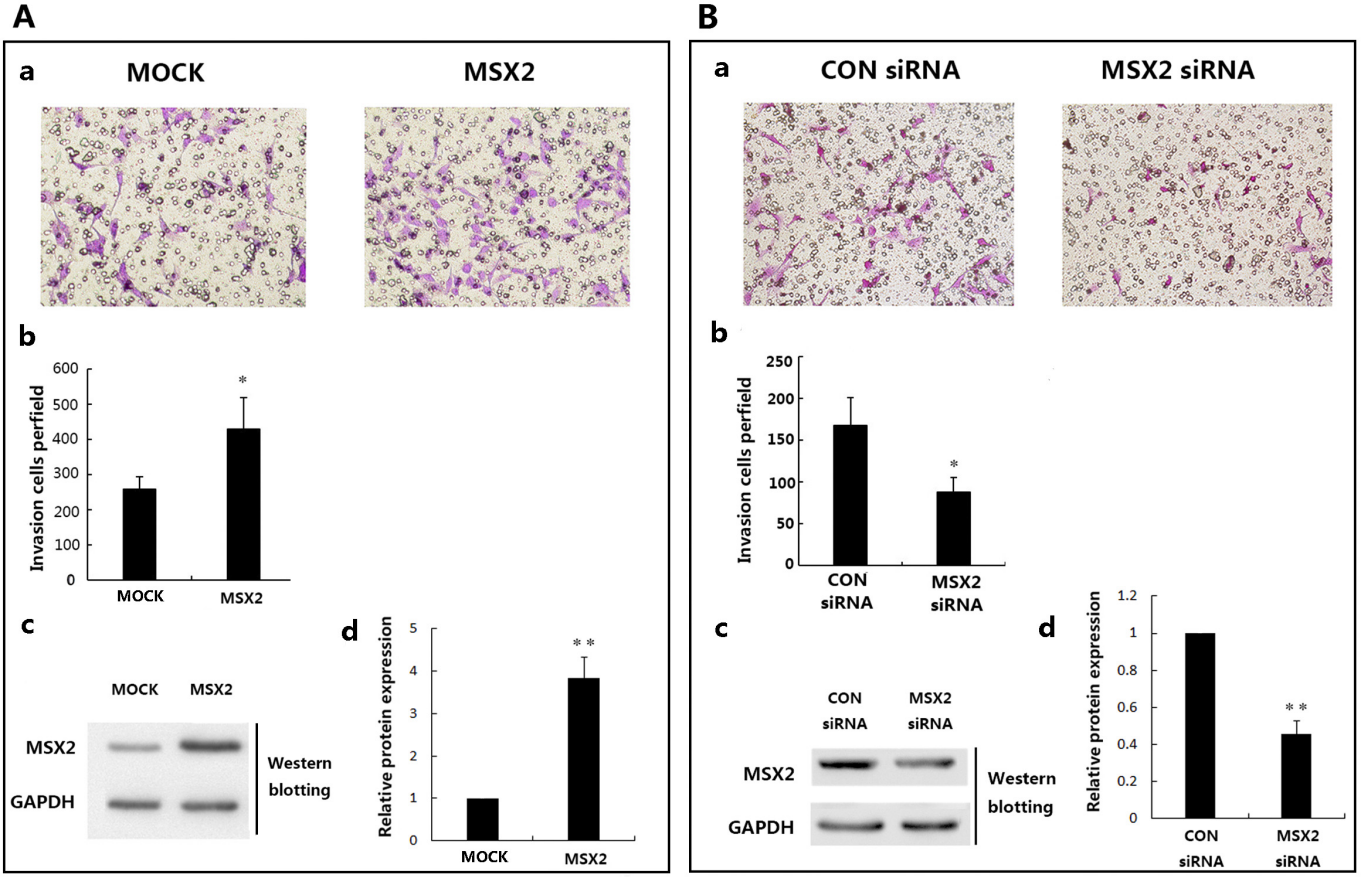
Overexpression and siRNA-mediated knockdown of MSX2 results in enhanced and reduced HTR8/SVneo cell invasion, respectively. **(A)** Overexpression of MSX2 induces invasion in HTR8/SVneo cells. (a) Representative images of filters containing invaded cells from Matrigel invasion assays. (b) Statistical bar graphs showing the averaged results from three independent experiments (*t*-test). (c) Examination of MSX2 overexpression efficiency after transfection. And statistical assay of the results in (d) (*t*-test; *P < 0.05). **(B)** siRNA-mediated knockdown of MSX2 expression inhibits HTR8/SVneo cell invasion. (a) Representative images of filters containing invaded cells from Matrigel invasion assays. (b) Statistical bar graphs showing the averaged results from three independent experiments. (*t*-test) (c) Examination of MSX2 knock down efficiency after siRNA transfection. And statistical assay of the results in (d) (*t*-test; **P < 0.01; n = 3). GAPDH was used as a loading control.

To investigate the role of MSX2 in trophoblast cell invasion, we utilized the human first trimester extravillous trophoblast cell line HTR8/SVneo. First, we successfully upregulated MSX2 expression(3.78±0.59 folds compared with MOCK) in these cells via transfection with a recombinant overexpression plasmid carrying the *MSX2* gene (Fig 2Ac and 2Ad). The effects of MSX2 expression on motility and invasion of HTR8/SVneo cells were evaluated using Matrigel invasion assays. As shown in Fig 2Aa and 2Ab, overexpression of MSX2 resulted in marked increases in the invasive ability of HTR8/SVneo cells compared to the control. Conversely, transfection of HTR8/SVneo cells with a MSX2-specific siRNA resulted in suppression of MSX2 expression (0.47±0.09 folds compared with CON siRNA) and significantly diminished invasive ability, compared with the control cell population, as determined by Matrigel invasion assay(Fig 2B, 2Ba and 2Bb) and western blot analyses, respectively (Fig 2Bc and 2Bd).

### Correlation between MSX2 expression and MMP activity

**Fig 3:**
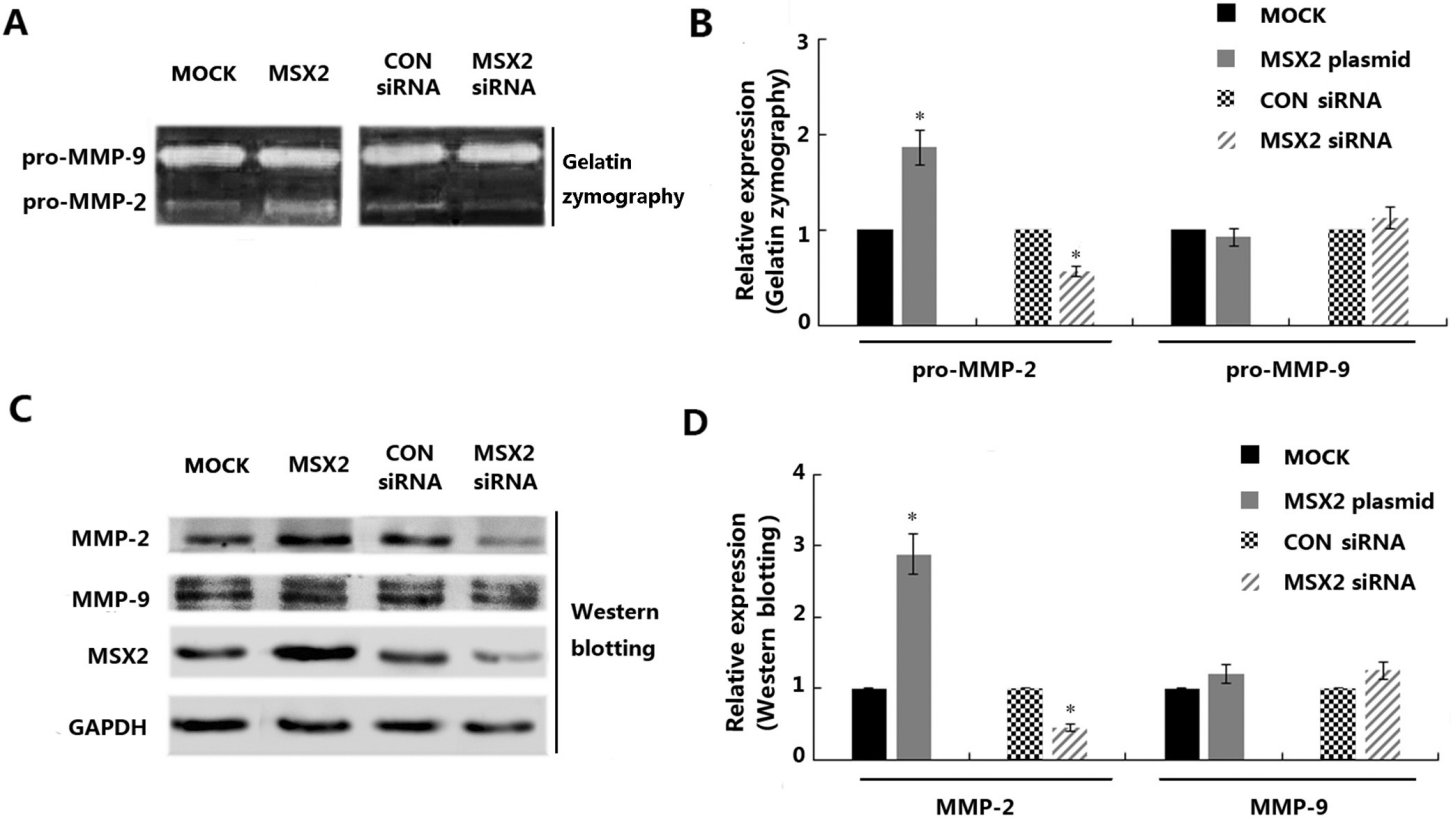
Modulation of MSX2 expression in HTR8/SVneo cells influences the gelatinolytic activities of pro-MMP-2 and MMP-2. **(A)** Serum-free culture medium was harvested from cells transfected with the MSX2 overexpression vector and the MSX2 siRNA, as well as from the control cell populations, and subjected to gelatin zymography assay analysis. **(B)** Graphical representation of the zymographic results shown in **A**. **(C)** HTR8/SVneo cells transfected with plasmids or siRNA molecules were subjected to western blot analysis using the indicated antibodies. **(D)** Graphic depiction of the western blot results (three independent experiments). GAPDH was used as a loading control for western blot analyses (Mann Whitney test; *P < 0.05; n = 3).

Gelatinases (MMP-2 and MMP-9) play pivotal roles in the extracellular matrix remodeling during trophoblast invasion. As such, we investigated the effects of MSX2 expression on the activities of MMP-2 and MMP-9 in HTR8/SVneo cells. For these analyses, supernatant and total protein samples were harvest at 48 h after transfection with plasmids or siRNA molecules, and subjected to gelatin zymography assay analysis. Overexpression of MSX2 resulted in significant increases in pro-MMP-2, but not pro-MMP-9, activity in the supernatants of HTR8/SVneo cells, compared with the control (*P*<0.05). In contrast, the supernatant harvested from cells treated with the MSX2-specific siRNA exhibited prominently decreased pro-MMP-2, but not pro-MMP-9, activity (Fig 3A and 3B). Consistent with these findings, western blot analysis revealed that HTR8/SVneo cells transfected with the MSX2 plasmid and the MSX2-specific siRNA exhibited increased and decreased MMP-2 expression compared with the control cells, respectively, but no changes in MMP-9 expression (Fig 3C and 3D, *P*<0.05).

### Modulation of MSX2 expression has no obvious effect on proliferation and apoptosis in HTR8/SVneo cells

**Fig 4:**
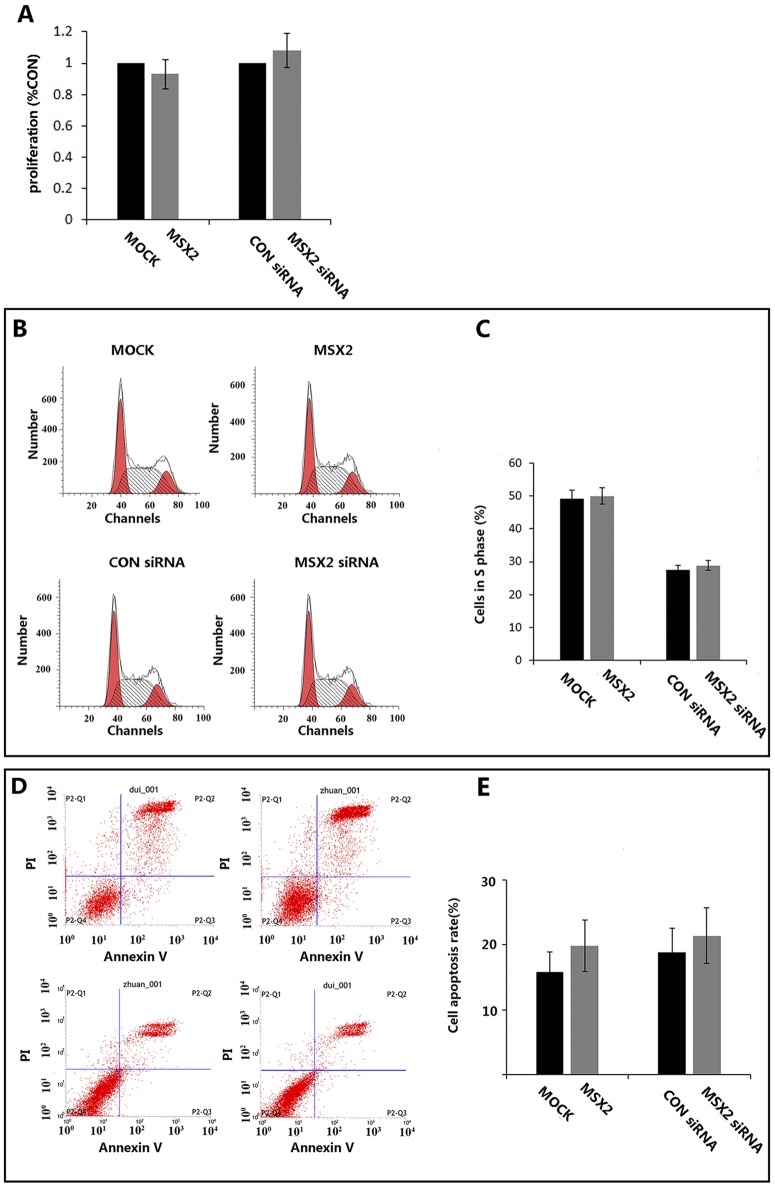
Effects of MSX2 overexpression or knockdown on proliferation and apoptosis of HTR8/SVneo cell. **(A)** MTT assay of HTR8/SVneo cell transfected with indicated vectors or siRNAs showed no significant difference on proliferation. (*t*-test). **(B, D)** Cell cycle progression was analyzed by flow cytometry at 24 h after transfection with the MSX2 overexpression vector or the MSX2 siRNA. Results were evaluated using MODFIT software. **(C, E)**Statistical bar graphs summarizing the results of three independent experiments in,respectively.(*t*-test,*P*>0.05; n = 3).

After Matrigel cell invasion assays, we further investigated the effects of MSX2 on trophoblast cell HTR8/SVneo proliferation and apoptosis. HTR8/SVneo cells transfected with the siRNA or the plasmids were subjected to MTT assay(Fig 4A) and subsequent flow cytometry analysis by PI staining(Fig 4B). Meanwhile, apoptosis levels were evaluated using a FITC-labeled annexin V-specific antibody (Fig 4D). Our results indicated that either MSX2 knock down or over expression had no obvious effects on cell proliferation or apoptosis compared with the control groups (*P*>0.05).

### Elevated MSX2 expression promotes EMT in trophoblast cells

**Fig 5:**
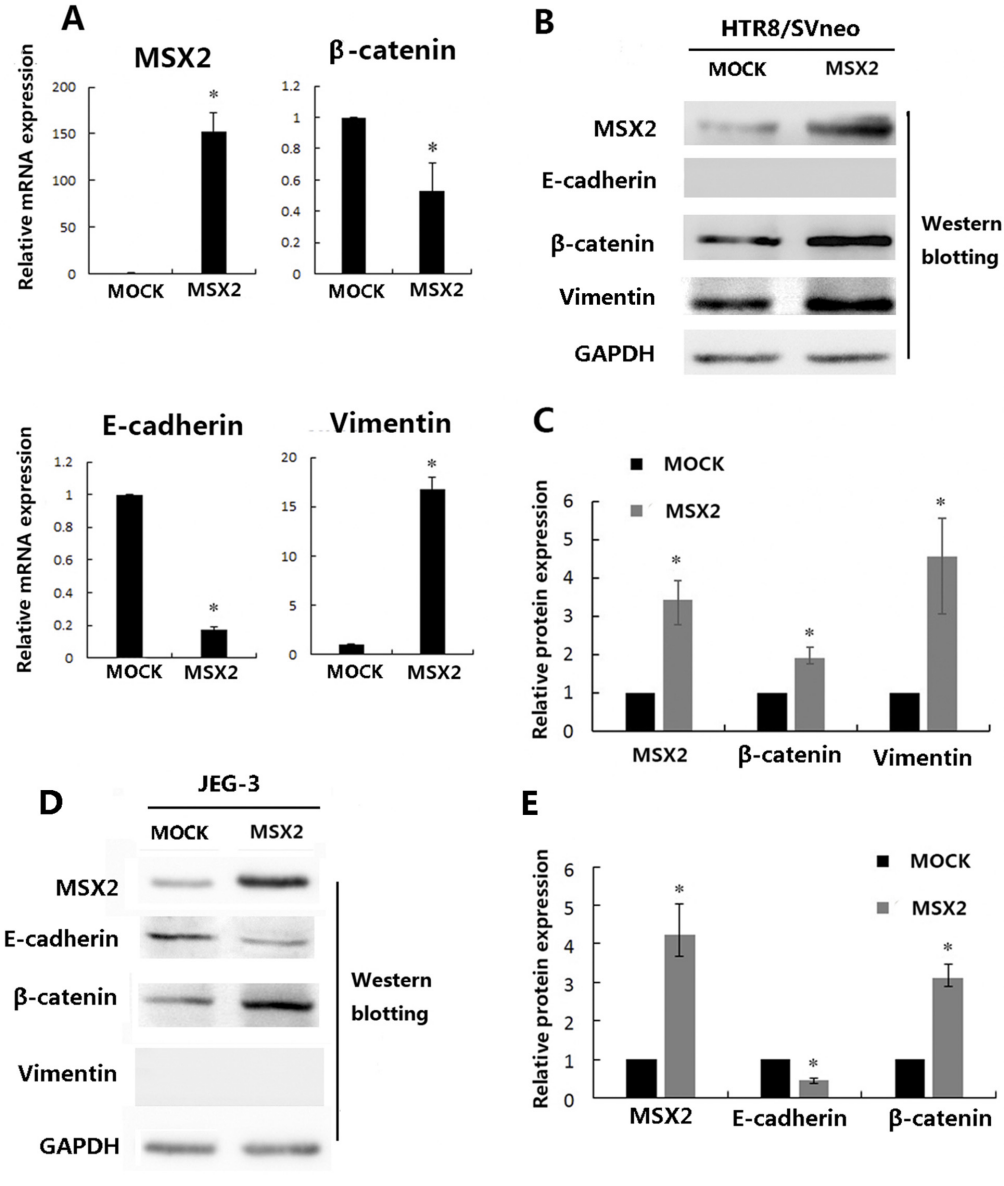
The effects of overexpression MSX2 on EMT markers in trophoblast cell lines. **(A)** Statistical analysis of mRNA expression levels in HTR8/SVneo cells after transfection with the indicated plasmids by quantitative reverse transcription (qRT)-PCR (Mann Whitney test; *P < 0.05). **(B)** HTR8/SVneo cells were transfected with the control or MSX2 expression plasmids and subjected to western blotting analysis using the indicated antibodies. And statistical assay from three independent experiments of the results in (**C**) (Mann Whitney test; *P < 0.05). **(D)** JEG-3 cells were transfected with the control or MSX2 expression plasmids and subjected to western blot analysis using the indicated antibodies. And statistical assay from three independent experiments of the results in (**E**) (Mann Whitney test; *P < 0.05).

**Fig 6:**
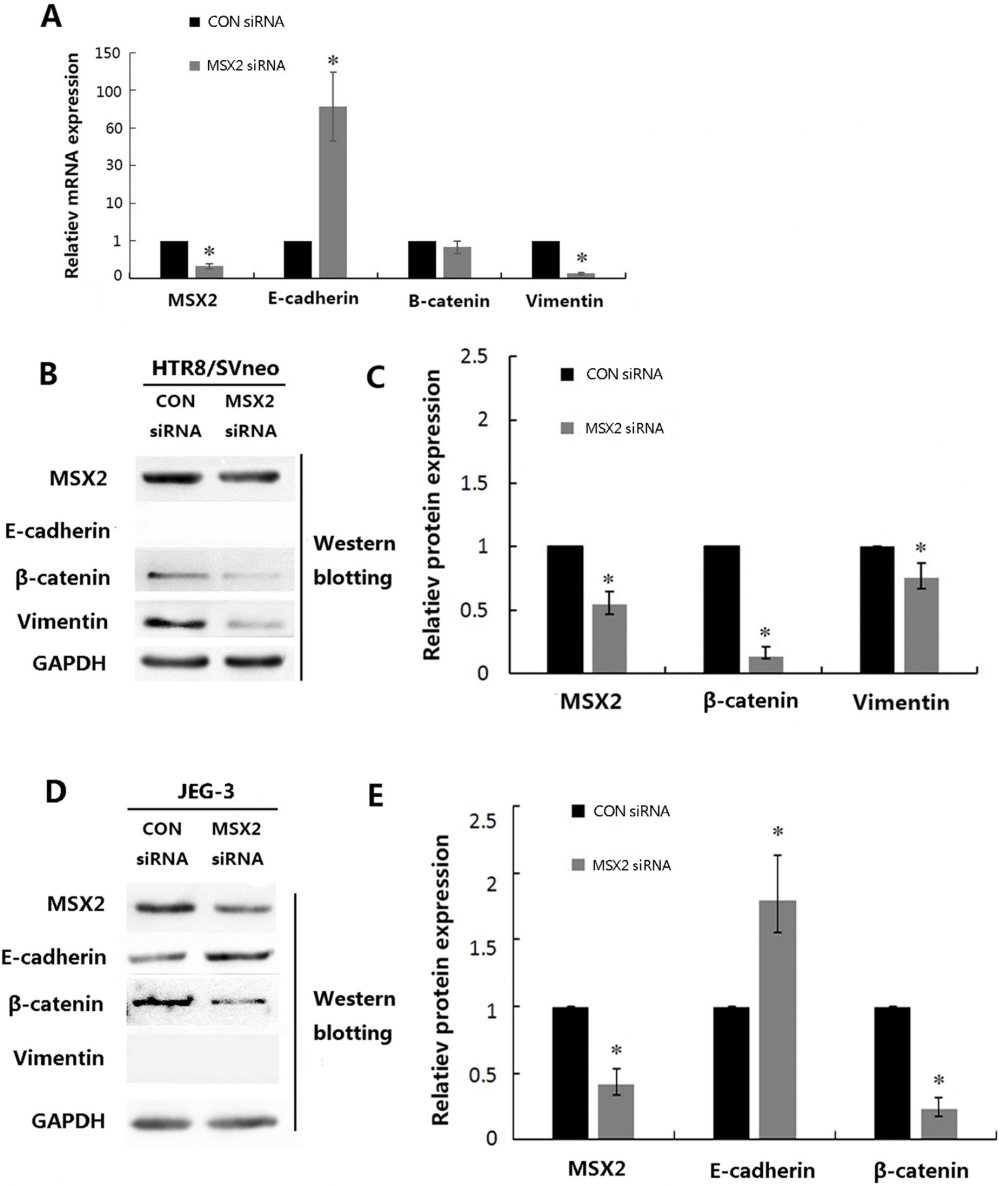
The effects of siRNA-mediated knockdown of MSX2 on EMT markers in trophoblast cell lines. **(A)** Statistical analysis of the mRNA expression levels of the indicated genes in HTR8/SVneo cells after transfection with the indicated siRNA molecules. **(B)** HTR8/SVneo cells were transfected with the indicated siRNAs and subjected to western blot analyses using the indicated antibodies. And statistical assay from three independent experiments of the results in (**C**) (Mann Whitney test; *P < 0.05). **(D)** JEG-3 cells were transfected with the indicated siRNAs and subjected to western blot analysis using the indicated antibodies. And statistical assay from three independent experiments of the results in (**E**) (Mann Whitney test; *P < 0.05).

Previous studies demonstrated that MSX2 promotes EMT through activation of the Wnt/β-catenin signaling pathway in cancer cells [10, 15, 16, 22]. We therefore investigated the effects of differential MSX2 on the expression of β-catenin and vimentin (mesenchymal makers), and of E-cadherin (epithelial maker) in HTR8/SVneo and JEG-3 cells, respectively. qRT-PCR (Fig 5A) and western blot (Fig 5B and 5C) analyses indicated that overexpression of MSX2 resulted in reduced mRNA expression of E-cadherin and enhanced mRNA and protein expression of vimentin in HTR8/SVneo cells (*P*<0.05). Interestingly, overexpression of MSX2 also resulted in reduced mRNA expression but increased protein expression of β-catenin in HTR8/SVneo cells. Consistent with these findings, siRNA-mediated knockdown of MSX2 resulted in reduced mRNA (Fig 6A) and protein (Fig 6B and 6C) expression of vimentin in HTR8/SVneo cells(*P*<0.05). Furthermore, while the protein expression levels of β-catenin markedly reduced in cells treated with the MSX2 siRNA (*P*<0.05), these cells exhibited no changes in β-catenin mRNA levels(*P*>0.05).

Similar to the results obtained in HTR8/SVneo cells, overexpression of MSX2 resulted in downregulation of E-cadherin and upregulation of β-catenin protein expression in the epithelial choriocarcinoma cell line JEG-3 (Fig 5D and 5E), while treatment with the MSX2 siRNA resulted in increased and decreased E-cadherin and β-catenin expression, respectively (Fig 6D and 6E). These findings suggest that MSX2 may induce EMT via activation of the Wnt/β-catenin pathway in both HTR8/SVneo and JEG-3 cells.

### MSX2 protein expression is downregulated in placental villi from PE patients

**Fig 7:**
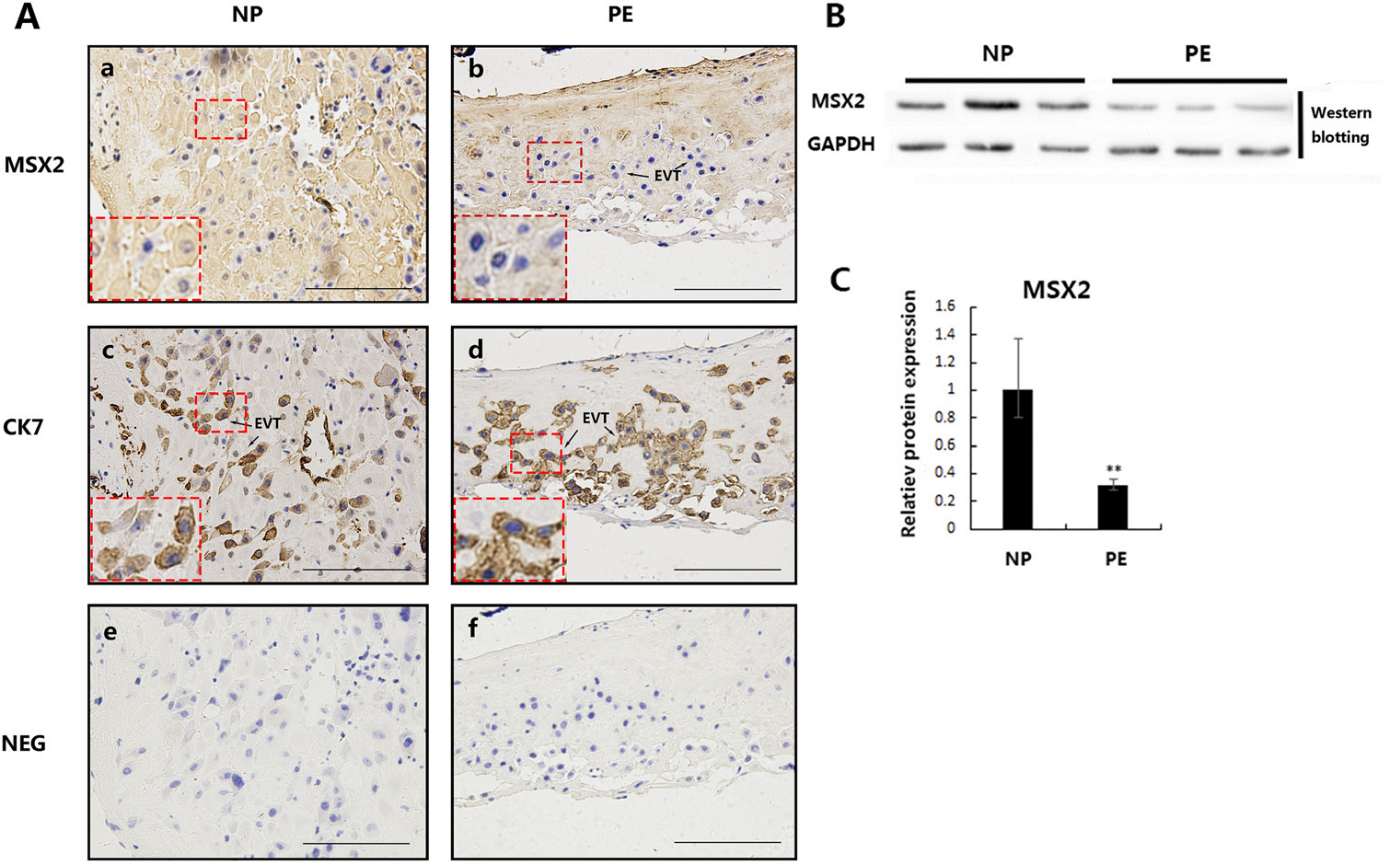
MSX2 is significantly downregulated in the placental villi from PE patients. **(A)** Immunostaining of MSX2 in the EVTs from normal and PE placentas at similar gestational age (a and b). CK7 was used as a marker for EVTs (c and d). Negative control images (NEG) in which normal IgG was used to replace the primary antibody showed no specific positive staining (e and f). Boxed region is enlarged on the under panel. Scale bar = 200μm. NP: nomal term placenta PE: pre-eclampsia **(B)** Western blot analysis of MSX2 expression in the human placental villi from term placentas (38 weeks; n = 3) and PE placentas (38 weeks; n = 3). **(C)** Results obtained in B were quantified by measuring the protein band intensity of MSX2 relative to that of GAPDH (n = 8; *t*-test; **P < 0.01).

Inadequate trophoblast migration/invasion and impaired spiral artery remodeling can result in poor placental perfusion leading to pregnancy-related diseases such as PE [23]. Placental villi from pre-eclamptic and normal pregnancy patients of close gestational stages were analyzed by immunohistochemistry (Fig 7A) and were dissected and subjected to western blot analysis (Fig 7B). Immunohistochemistry showed weak positive signals for MSX2 in the EVTs from PE placentas (Fig 7Ab) compared with the strong MSX2 staining in the EVTs from normal placentas (Fig 7Aa). Western blotting(Fig 7B) and statistical analyses o(Fig 7C) indicated that MSX2 was expressed at significantly lower levels in the placental villi from pre-eclamptic patients than in those from control placentas (n = 8).

## Discussion

In the present study, we utilized immunohistochemistry analysis to examine the expression patterns of MSX2 in placental villi from three stages of human pregnancy and in trophoblast-derived cells in vitro. The significant levels of MSX2 expression observed in CTB, STB, and EVT cells suggest a putative role for this protein in trophoblastic invasion during embryo implantation. Consistent with this conclusion, MSX2 protein levels were markedly higher in the choriocarcinoma-derived JEG-3 and JAR cell line than in the normal trophoblast-derived HTR8/SVneo cell line. We therefore evaluated this hypothesis using a Matrigel invasion model and HTR8/SVneo cells. As expected, the invasive ability of trophoblast cells was enhanced by MSX2 overexpression, which was accompanied by upregulation of pro-MMP-2 and MMP-2, and was inhibited by siRNA-mediated downregulation of MSX2 expression. These data therefore indicate that MSX2 plays an important role in human placental trophoblast invasion. Notably, MSX2 overexpression also resulted in downregulation of E-cadherin and upregulation of both vimentin and β-catenin, while MSX2 knockdown yielded upregulation of E-cadherin and downregulation of vimentin and β-catenin. Collectively, these findings indicate that MSX2 expression may promotes the EMT process.

As mentioned above, MSX2 may promote trophoblastic invasion via activation of the EMT process. Intriguingly, the mRNA expression levels of β-catenin were inconsistent with those of the protein in HTR8/SVneo cells transfected with the MSX2 overexpression plasmid. From these results, we infer that MSX2 does not directly influence the mRNA expression level of β-catenin. Instead, through activation of canonical Wnt signaling pathways, we hypothesis that MSX2 may stabilize β-catenin protein expression and increases its cytoplasmic concentration by disrupting the APC/Axin/GSK-3β/CK1α destruction complex, which is known to degrade cytosolic β-catenin[24]. Subsequently, the active, dephosphorylated β-catenin translocates into the nucleus where it binds to specific transcription factors, thereby promoting the expression of numerous genes that regulate developmental processes, cell cycle progression, cell differentiation, and cell invasion, which are all closely dependent on EMT events [25, 26]. Hence, It is conceivable the downregulation of β-catenin mRNA after MSX2 overexpression might be due to the negative feedback loop resulting from redundant cytoplasmic β-catenin protein.

Since MSX2 promotes trophoblastic invasion via EMT, we speculated that MSX2 might play a role in PE pathology, which could be associated with shallow invasion of trophoblast cells[27]. As expected, we observed lower levels of MSX2 expression in the placental villi from pre-eclamptic patients than that in those from normal placentas by immunohistochemistry and western blot assays. However, the mechanism governing the modulation of MSX2 expression in PE placental tissues remains unclear. Likewise, whether Wnt/β-catenin signaling is involved in this process requires further investigation.

Several studies have demonstrated that MSX2 plays an important role in tissuedevelopment. BMP4-induced MSX2 expression was shown to promote EMT in human embryonic stem cells, which subsequently differentiate into the mesoderm and then preferentially toward the cardiovascular lineage [17]. In a separate study on dentinogenesis, upregulation of MSX2 resulted in decreased expression of DDK1, an inhibitor of the Wnt signaling pathway [28]. In addition, while earlier reports detected elevated MSX2 expression in a variety of carcinoma cell lines, more recent studies demonstrated that MSX2 promotes EMT through activation of Wnt signaling in cancer cells [10, 15, 16, 22]. Moreover, activation of the Wnt pathway via treatment with a Wnt3a ligand or a GSK3β inhibitor resulted in strong induction of MSX2 expression in ovarian cancer cells [14]. To date, however, the molecular mechanism by which MSX2 regulates Wnt signaling remains largely unclear.

Recent evidence indicates that activation of the Wnt/β-catenin pathway is necessary for differentiation of fetal CTBs into an invasive phenotype during placentation [29]. Similar to MSX2, β-catenin is expressed predominantly in STB and EVT cells. Furthermore, the expression of this protein is significantly decreased in pre-eclamptic placental tissues compared to normal placental controls [30]. Canonical Wnt signaling through β-catenin is critical for blastocyst-uterine communication, subsequent implantation, and placental development and differentiation; however, overexpression of this protein can lead to rare placental carcinomas [31]. In mice, canonical Wnt signaling is dynamically activated during two distinct stages: initially during the development of the circular smooth muscle of the myometrium, followed by activation in the luminal epithelium directly opposed to the blastocyst at implantation sites, both of which require the presence of a blastocyst [32]. Notably, inhibition of Wnt/β-catenin signaling prevents implantation in mice [32]. Methylation of certain genes encoding inhibitors of Wnt/β-catenin signaling has been detected in human placentas and trophoblast cells, supporting a role for Wnt/β-catenin signaling in trophoblast invasion [33]. Interestingly, elevated methylation of the promoter for the gene encoding adenomatous polyposis coli (APC), an inhibitor of β-catenin, was detected in choriocarcinoma trophoblast cell lines [34]. While Wnt3a promotes trophoblast invasion, this effect is blocked by Dickkopk1 (DKK1), an inhibitor of the canonical Wnt signaling pathway [35]. Additional studies detected enhanced expression of Wnt signaling pathway components in first trimester and term human placental tissues, as well as in several trophoblast cell lines [26, 36]. Furthermore, the canonical Wnt signaling pathway ligand Wnt3a was previously reported to promote motility and invasiveness of an EVT cell line and of primary EVTs purified from first-trimester placentas through activation of the Wnt/β-catenin signaling pathway and upregulation of MMPs [29, 37]. Lastly, both Wnt2 and β-catenin expression are decreased, while DKK1 expression is increased in pre-eclampsic placentas [30]. In summary, canonical Wnt activity has been shown to promote trophoblast invasion, with hyperactivation leading to trophoblast disorders such as choriocarcinoma and hydatidiform moles, while downregulation of the Wnt signaling cascade can be found in PE [38].

Recently, a study on early pregnancy indicated that expression of MSX2, one of the 13 tightly interconnected transcription factors of the trophectoderm core transcriptional regulatory circuitry, is induced in the trophectoderm and maintained in the placenta [39]. Furthermore, using mutant mouse models harboring conditional deletions of the gene(s) encoding MSX1 and/or MSX2 in the uterus, MSX1 and MSX2 were found to play critical roles in regulating uterine function during implantation. Specifically, loss of MSX1/MSX2 expression correlated with altered uterine luminal epithelial cell polarity, and affected E-cadherin/β-catenin complex formation through modulation of Wnt5a expression [19].

To date, there is no published evidence indicating a correlation between MSX2 expression and trophoblast development. In the current study, we examined the effect of MSX2 expression on trophoblastic invasion. Our results suggest that MSX2 may activates the Wnt/β-catenin signaling pathway to promote EMT events. However, further studies are required to elucidate the specific associations between MSX2 and factors such as Wnt3a and DDK1, and the collaboration of MSX2 with Wnt/β-catenin signaling pathway in trophoblast cell behaviors. Meanwhile, another MSX family member, MSX1, has been shown to function similarly to or to compensate for a lack of MSX2 in many aspects[18, 19, 40, 41]. Using an immunofluorescence microscopy approach, we found that MSX1 co-localizes with MSX2 in villi trophoblast cells (data not shown); however, the effects of MSX1 expression on trophoblast cell behaviors require further analysis. In conclusion, the results of this study provide insight into the mechanism of trophoblastic invasion and may be helpful to elucidate the mechanism of PE pathogenesis.
